# Whole grain consumption and risk of colorectal cancer: a population-based cohort of 60 000 women

**DOI:** 10.1038/sj.bjc.6602543

**Published:** 2005-04-12

**Authors:** S C Larsson, E Giovannucci, L Bergkvist, A Wolk

**Affiliations:** 1 Division of Nutritional Epidemiology, The National Institute of Environmental Medicine, Karolinska Institutet, Box 210, SE-17177 Stockholm, Sweden; 2 Departments of Nutrition and Epidemiology, Harvard School of Public Health, 677 Huntington Avenue, Boston, MA 02115, USA; 3 Channing Laboratory, Department of Medicine, Brigham and Women's Hospital and Harvard Medical School, 181 Longwood Avenue, Boston, MA 02115, USA; 4 Department of Surgery and Centre for Clinical Research, Central Hospital, SE-72189 Västerås, Sweden

**Keywords:** cohort studies, colon cancer, epidemiology, dietary fibre, rye, whole grains

## Abstract

We examined prospectively the association between whole grain consumption and colorectal cancer risk in the population-based Swedish Mammography Cohort. A total of 61 433 women completed a food-frequency questionnaire at baseline (1987–1990) and, through linkage with the Swedish Cancer Registry, 805 incident cases of colorectal cancer were identified during a mean follow-up of 14.8 years. High consumption of whole grains was associated with a lower risk of colon cancer, but not of rectal cancer. The multivariate rate ratio (RR) of colon cancer for the top category of whole grain consumption (⩾4.5 servings day^−1^) compared with the bottom category (<1.5 servings day^−1^) was 0.67 (95% confidence interval (CI), 0.47–0.96; *P*-value for trend=0.06). The corresponding RR after excluding cases occurring within the first 2 years of follow-up was 0.65 (95% CI, 0.45–0.94; *P*-value for trend=0.04). Our findings suggest that high consumption of whole grains may decrease the risk of colon cancer in women.

About three decades ago, Burkitt (1971) put forth the hypothesis that the refining of grains and the lack of dietary fibre in the diet may be implicated in colorectal carcinogenesis. In addition to being a concentrated source of dietary fibre and certain vitamins and minerals, whole grains contain various phytochemicals that may have anticancer properties (Slavin *et al*, 1999).

In a review and meta-analysis, nine out of 10 case–control studies reported an inverse association between whole grain consumption and risk of colorectal cancer or adenoma; the summary odds ratio for the highest compared with the lowest category of consumption was 0.79 (Jacobs *et al*, 1998). Two cohort studies conducted in the United States (McCullough *et al*, 2003) and Finland (Pietinen *et al*, 1999) reported no association between overall whole grain consumption and risk of colorectal or colon cancer. However, in the Alpha-Tocopherol, Beta-Carotene Cancer Prevention Study among Finnish male smokers, there was a nonsignificant reduction in colorectal cancer risk associated with a high consumption of rye products (Pietinen *et al*, 1999).

Given the paucity of prospective data on the association between consumption of whole grains and colorectal cancer risk, we examined this relationship among 61 433 women in the population-based Swedish Mammography Cohort. This cohort of Swedish women allowed us to assess consumption of hard whole grain rye bread – a concentrated source of rye fibre and related factors – in relation to colorectal cancer incidence.

## SUBJECTS AND METHODS

### Study population

The population-based Swedish Mammography Cohort was established between 1987 and 1990, when all women who were 40–76 years of age and resided in Uppsala or Västmanland Counties in central Sweden received a mailed questionnaire regarding diet, weight, height, and education. Among 90 303 women in the source population, 66 651 (74%) responded to the questionnaire. An expanded questionnaire that elicited information on lifestyle factors, medications, and smoking history was sent to participants in 1997.

For this analysis, we excluded women with missing date on the baseline questionnaire, women with an erroneous National Registration Number, and women who reported extreme total energy intakes (i.e., 3 standard deviations from the mean value for log-transformed energy). In addition, we excluded women with a diagnosed cancer (other than nonmelanoma skin cancer) at baseline, leaving 61 433 women eligible for this analysis. The investigation was approved by the Ethics Committees at the Uppsala University Hospital (Uppsala) and the Karolinska Institutet (Stockholm).

### Dietary assessment

Dietary information was derived from a 67-item food-frequency questionnaire administered at baseline. We asked participants to indicate their average consumption of each food item during the previous 6 months. Participants could choose from eight frequency categories, ranging from never/seldom to four or more times per day. Whole grain foods included hard whole grain rye bread (such as Wasa bread), soft whole grain bread, porridge, and cold breakfast cereals. Refined grains included soft white bread, pasta, rice, pancakes or waffles, and sweet buns or biscuits. We computed nutrient intakes by multiplying the consumption frequency of each food item by the nutrient content of age-specific (<53, 53–65, ⩾66 years) portion sizes. Values for nutrients in foods were obtained from the Swedish National Food Administration Database (Bergström *et al*, 1991). All nutrients and cereal fibre were energy-adjusted using the residual method (Willett and Stampfer, 1986). In a validation study among 129 women randomly selected from the cohort, Spearman correlation coefficients between data from four 1–week diet records and the food-frequency questionnaire were 0.5 for hard whole grain rye bread, 0.5 for soft whole grain bread, 0.6 for porridge, and 0.7 for cold breakfast cereals.

### Case ascertainment and follow-up of the cohort

The National Swedish Cancer Registry provided data until 31 December 2002; additional information until end of follow-up (30 June 2004) was obtained from the Regional Cancer Registry covering the study area. Follow-up for cancers through these registries is nearly 100% complete (Mattsson and Wallgren, 1984). Colon cancers were considered to be those located above the peritoneal delineation of the abdominal cavity, and rectal cancers were those occurring below this delineation. Tumours originating from the caecum through splenic flexure were considered proximal colon cancers, and those in the descending and sigmoid colon were considered distal colon cancers. We identified deaths in the cohort and the date when a participant moved out from the study area by matching with the Swedish Death and Population Registers.

### Statistical analysis

For each participant, follow-up time accrued from the date of entry into the cohort and ended at the date of a colorectal cancer diagnosis, the date of death, the date of leaving the study area, or 30 June 2004, whichever came first. Participants were grouped into five roughly equal categories according to their frequency of whole grain consumption and into quintiles according to their cereal fibre intake. Rate ratios (RRs) of colorectal cancer for each of the upper categories were calculated by dividing the incidence rates in these categories by the incidence rate in the lowest category.

The proportional hazards assumptions were satisfied, and we estimated the RRs with 95% confidence intervals (CIs) using Cox proportional hazards models (Cox and Oakes, 1984) stratified on age in months at baseline and the year of entry into the cohort. In multivariate models, we simultaneously controlled for age, body mass index, education, and intakes of total energy, saturated fat, calcium, red meat, fruits, and vegetables. In additional analyses, we used information from the 1997 questionnaire to further adjust for family history of colorectal cancer, cigarette smoking, physical activity, and use of aspirin and multivitamin supplements. Tests for linear trend were conducted using the median value for each exposure category analysed as a continuous variable. All analyses were conducted with SAS software (version 9.1; SAS Institute Inc., Cary, NC, USA), and all *P*-values were two sided.

## RESULTS

At baseline in 1987–1990, the mean daily whole grain consumption was 2.6 servings. Whole grain consumption varied considerably, with median daily consumption in the top category being 5.0 servings compared with 1.1 servings in the bottom category (Table 1). Women with a high consumption of whole grains were older, leaner, and more likely to have a postsecondary education. Greater consumption of whole grains was also associated with higher consumption of fruits and vegetables, but with a lower intake of saturated fat.

During a mean follow-up of 14.8 years (911 042 person-years), we ascertained 805 incident cases of colorectal cancer, including 547 colon cancers (249 proximal colon, 170 distal colon, and 128 unspecified) and 252 rectal cancers; six cases were diagnosed with both colon and rectal cancer. We observed an inverse association between consumption of whole grains and risk of colon cancer, but not of rectal cancer (Table 2). Compared with women in the lowest category of whole grain consumption (<1.5 servings day^−1^), the age-adjusted RR of colon cancer for women in the highest category (⩾4.5 servings day^−1^) was 0.67 (95% CI, 0.49–0.91; *P*-value for trend=0.03). The RR was virtually unchanged after further adjustment for education, body mass index, and intakes of total energy, saturated fat, calcium, red meat, fruits, and vegetables (RR, 0.67; 95% CI, 0.47–0.97). Additional controlling for alcohol consumption, family history of colorectal cancer, smoking, physical activity, and use of aspirin and multivitamins did not alter the findings essentially (RR, 0.69; 95% CI, 0.48–0.99). We conducted further analyses after excluding cases that occurred during the first 2 years of follow-up (Table 2). The association of whole grain consumption with colon cancer risk became slightly stronger; compared with women in the bottom category of whole grain consumption, those in the top category had a significant 35% lower risk. High consumption of whole grains was associated with a lower risk of both proximal colon (RR, 0.69; 95% CI, 0.40–1.20) and distal colon cancer (RR, 0.54; 95% CI, 0.27–1.08).

In analyses of individual whole grain foods, only hard whole grain rye bread had a statistically significant inverse association with colon cancer risk. After excluding cases that occurred within 2 years of follow-up, the multivariate RR of colon cancer for women who consumed two or more slices per day of hard whole grain bread compared with those who consumed less than four slices per week was 0.74 (95% CI, 0.55–0.98; *P*-value for trend=0.02). Each daily increment of one slice of hard whole grain bread was associated with a 12% reduction in colon cancer risk (multivariate RR, 0.88; 95% CI, 0.78–0.99).

Refined grain consumption was not appreciably associated with risk of colon or rectal cancer. The multivariate RRs for the highest (⩾2.5 servings day^−1^) compared with the lowest category (<0.5 servings day^−1^) of refined grain consumption were 1.29 (95% CI, 0.92–1.82) for colon cancer and 0.82 (95% CI, 0.48–1.40) for rectal cancer.

**Figure 1:**
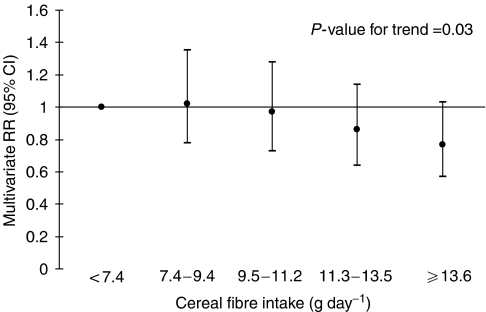
Multivariate RR and 95% CI of colon cancer according to cereal fibre intake. Multivariate RRs were adjusted for age, body mass index (quartiles), education (less than high school, high school, or university), total energy intake (continuous), and quartiles of intakes of saturated fat, calcium, red meat, fruits, and vegetables. RR=rate ratios; CI=confidence intervals.

We also examined the association between cereal fibre intake and risk of colon cancer (Figure 1). After controlling for age, education, body mass index, and intakes of total energy, saturated fat, calcium, red meat, fruits, and vegetables, women in the top quintile of cereal fibre intake had a 27% (RR, 0.77; 95% CI, 0.57–1.03) reduced risk of colon cancer compared with those in the bottom quintile. Further adjustment for intakes of vitamin C, vitamin E, *β*-carotene, folate, vitamin B_6_, and magnesium did not materially change the results, although the confidence interval widened (RR, 0.76; 95% CI, 0.53–1.08). There was no association of cereal fibre intake with rectal cancer risk (RR, 0.99; 95% CI, 0.64–1.53).

To elucidate whether the inverse relation between whole grain consumption and colon cancer risk could be attributed to cereal fibre, we included intakes of whole grains and cereal fibre simultaneously in a multivariate model (adjusted for the same variables as in Table 2). In this model, the RR of colon cancer comparing the extreme categories of whole grain consumption was attenuated from 0.65 to 0.75 (95% CI, 0.49–1.15).

## DISCUSSION

In this large population-based prospective cohort of women, a high consumption of whole grain foods was associated with a lower risk of colon cancer. The decrease in risk was 35% when comparing the extreme categories of whole grain consumption, and this was reduced to 25% after additional adjustment for cereal fibre. This suggests that the observed reduction in colon cancer risk associated with high consumption of whole grains may partly be attributed to cereal fibre but that other constituents may contribute to further protection.

There are several components of whole grains that may reduce the risk of colon cancer, including various vitamins (especially B-vitamins) and minerals (e.g. magnesium and zinc), phenolic compounds, antinutrients (e.g. phytic acid, tannins, and enzyme inhibitors), and phyto-oestrogens (Slavin *et al*, 1999). Whole grains are also a rich source of dietary fibre, resistant starch, and oligosaccharides that can influence the gut environment. Insoluble fibre in grains increases the bulk of luminal contents, thereby diluting potential carcinogens and promoters in the colon and decreasing transit time, and, consequently, reduces the exposure of the colonic epithelium to harmful compounds (Slavin *et al*, 1999). Although an inverse association between dietary fibre intake and colon cancer risk has been observed in a number of case–control studies (Kushi *et al*, 1999), large cohort studies have provided inconclusive findings. While no association between dietary fibre and colorectal cancer risk was observed in cohort studies in the United States (Giovannucci *et al*, 1994; Steinmetz *et al*, 1994; Fuchs *et al*, 1999), the European Prospective Investigation into Cancer and Nutrition (EPIC) (Bingham *et al*, 2003) among over 500 000 participants from 10 European countries reported significant inverse associations between intake of fibre from cereals and fruits and risk of colon cancer. A high intake of cereal fibre was also associated with a significant lower risk of colon adenomas in the Prostate, Lung, Colorectal, and Ovarian (PLCO) Cancer Screening Trial (Peters *et al*, 2003). The EPIC and PLCO studies observed no association between dietary fibre intake and risk of rectal cancer, which is consistent with our results.

Our findings for consumption of hard whole grain rye bread, a rich source of rye fibre, are consistent with those reported by Pietinen *et al* (1999) in Finland. In that Finnish cohort with 185 colorectal cancer cases among male smokers, the risk of colorectal cancer was 30% lower among men in the highest quartile of rye product consumption compared with those in the lowest quartile, but the findings were not significant (Pietinen *et al*, 1999). Rye bran is a rich source of lignans (Adlercreutz, 2002). Animal studies have suggested that rye bran and lignans may reduce colon carcinogenesis (Davies *et al*, 1999; Mutanen *et al*, 2000; Adlercreutz, 2002). It has been shown that rye bran more effectively suppresses colon carcinogenesis than wheat and oat bran (Mutanen *et al*, 2000).

Our study has the advantage of a large population-based sample size, a large number of colorectal cancer cases, and a virtually complete ascertainment of cancer cases (Mattsson and Wallgren, 1984). The prospective nature of our study precluded potential recall and selection biases, which could be an issue in case–control studies.

There are also potential limitations of our findings. It is likely that some measurement error existed in our estimation of whole grain consumption. However, measurement errors and resulting misclassification are likely to be unrelated to colorectal cancer in a prospective study, and would thus tend to weaken any association between whole grain consumption and colon cancer risk rendering our risk estimates conservative. As in any observational study, our results could be influenced, at least in part, by differences between participants in factors other than whole grain consumption. In general, women with a high consumption of whole grains consumed more fruits and vegetables, which may reflect a generally healthy lifestyle. Nevertheless, the observed inverse association between whole grain consumption and colon cancer remained essentially unchanged in multivariate models that accounted for other dietary and lifestyle factors, which argues against the possibility of residual confounding from these factors.

In conclusion, results from this large prospective population-based cohort of women provide support that high consumption of whole grain foods, particularly hard whole grain rye bread, may reduce the risk of colon cancer. This finding is consistent with the dietary recommendations to increase whole grains consumption.

## Figures and Tables

**Table 1:** Age-standardised baseline characteristics according to consumption of whole grains^a^

	**Categories of whole grain consumption (servings day^−1^)**				
**Characteristics**	**<1.5**	**1.5–2.4**	**2.5–3.4**	**3.5–4.4**	**⩾4.5**
Median consumption (servings day^−1^)	1.1	2.0	2.9	3.7	5.0
Mean age at baseline (years)	52.7	53.3	53.8	54.4	55.4
Mean body mass index (kg m^−2^)	25.0	24.8	24.6	24.6	24.2
Education ⩾12 years (%)	10.0	13.1	13.3	13.3	15.2

*Dietary intake*					
Cereal fibre (mg day^−1^)^b^	7.9	10.7	11.5	12.2	12.7
Saturated fat (g day^−1^)^b^	19.0	17.6	17.5	17.4	16.9
Calcium (mg day^−1^)^b^	699	702	702	693	705
Fruits (servings day^−1^)	1.2	1.5	1.6	1.6	1.8
Vegetables (servings day^−1^)	1.4	1.7	1.8	1.9	2.1
Red meat (servings week^−1^)	2.9	3.0	3.0	3.1	3.1

a Whole grains include hard whole grain rye bread, soft whole grain bread, porridge, and cold breakfast cereals.

b Nutrients adjusted to the rounded mean energy intake (1350 kcal day^−1^) in the cohort at baseline.

**Table 2:** RR and 95% CI of colorectal cancer according to consumption of whole grains

	**Categories of whole grain consumption (servings day^−1^)**					
	**<1.5**	**1.5–2.4**	**2.5–3.4**	**3.5–4.4**	**⩾4.5**	***P*-value for trend**
*Colorectal cancer*						
All cases						
Number of cases	187	201	191	132	94	
Age-adjusted RR (95% CI)	1.00	0.99 (0.81–1.22)	1.05 (0.85–1.29)	0.97 (0.77–1.22)	0.80 (0.62–1.03)	0.14
Multivariate RR (95% CI)^a^	1.00	1.00 (0.81–1.24)	1.05 (0.84–1.30)	0.96 (0.75–1.23)	0.80 (0.60–1.06)	0.16
Excluding cases with follow-up <2 years						
Number of cases	176	187	175	119	84	
Multivariate RR (95% CI)^a^	1.00	0.99 (0.80–1.23)	1.02 (0.81–1.29)	0.93 (0.72–1.21)	0.76 (0.56–1.03)	0.10

*Colon cancer* b						
All cases						
Number of cases	136	135	131	88	57	
Age-adjusted RR (95% CI)	1.00	0.92 (0.72–1.16)	1.00 (0.78–1.28)	0.90 (0.68–1.18)	0.67 (0.49–0.91)	0.03
Multivariate RR (95% CI)^a^	1.00	0.94 (0.73–1.21)	1.02 (0.79–1.33)	0.91 (0.67–1.23)	0.67 (0.47–0.96)	0.06
Excluding cases with follow-up <2 years						
Number of cases	129	125	124	79	52	
Multivariate RR (95% CI)^a^	1.00	0.91 (0.70–1.18)	1.02 (0.78–1.34)	0.86 (0.63–1.18)	0.65 (0.45–0.94)	0.04

*Rectal cancer* b						
All cases						
Number of cases	50	66	57	43	36	
Age-adjusted RR (95% CI)	1.00	1.22 (0.84–1.78)	1.14 (0.77–1.68)	1.17 (0.77–1.78)	1.16 (0.75–1.80)	0.61
Multivariate RR (95% CI)^a^	1.00	1.17 (0.80–1.72)	1.09 (0.72–1.64)	1.09 (0.69–1.72)	1.11 (0.67–1.83)	0.85
Excluding cases with follow-up <2 years						
Number of cases	46	62	48	39	31	
Multivariate RR (95% CI)^a^	1.00	1.21 (0.81–1.81)	1.02 (0.66–1.58)	1.11 (0.69–1.79)	1.07 (0.62–1.82)	0.99

a Multivariate RRs were adjusted for age, body mass index (quartiles), education (less than high school, high school, or university), total energy intake (continuous), and quartiles of intakes of saturated fat, calcium, red meat, fruits, and vegetables.

b Six cases diagnosed with both colon and rectal cancer are excluded from subsite-specific analysis. RR=rate ratio; CI=confidence interval.
